# Increased methylation at differentially methylated region of *GNAS* in infants born to gestational diabetes

**DOI:** 10.1186/s12881-014-0108-3

**Published:** 2014-10-01

**Authors:** Danqing Chen, Aiping Zhang, Min Fang, Rong Fang, Jiamei Ge, Yuan Jiang, Hong Zhang, Cong Han, Xiaoqun Ye, Hefeng Huang, Yun Liu, Minyue Dong

**Affiliations:** Women’s Hospital, School of Medicine, Zhejiang University, 1 Xueshi Road, Hangzhou, 310006 Zhejiang Province China; Bio-X Institutes, Key Laboratory for the Genetics of Developmental and Neuropsychiatric Disorders, Ministry of Education, Shanghai Jiao Tong University, Shanghai, China; Shaoxing Women and Children’s Hospital, Shaoxing, China; Huzhou Maternity and Child Care Hospital, Huzhou, China; Jiaxing Maternity and Child Care Hospital, Jiaxing, China; Ningbo Women and Children’s Hospital, Ningbo, China; Institutes of Biomedical Sciences, Fudan University, Shanghai, China; Department of Biochemistry and Molecular Biology, Key Laboratory of Molecular Medicine, The Ministry of Education, Fudan University Shanghai Medical College, 303 Mingdao Building, 138 Yixueyuan Road, Shanghai, 200032 PR China; Key Laboratory of Reproductive Genetics, Ministry of Education, Zhejiang University, Hangzhou, China; Key Laboratory of Women’s Reproductive Health of Zhejiang Province, Hangzhou, China

**Keywords:** Gestational diabetes mellitus, Methylation, DNA, DMR, *GNAS*, *IGF2*

## Abstract

**Background:**

Offspring of pregnancy complicated with gestational diabetes (GDM) are at high risk for metabolic diseases. The mechanisms behind the association of intrauterine exposure to GDM and high risk of health problems in later life remain largely unknown. The aim of this study was to clarify the alteration in methylation levels at differentially methylated regions (DMRs) of *GNAS* and *IGF2* in fetuses of GDM women and to explore the possible mechanisms linking maternal GDM with high risk of metabolic diseases in later life of GDM offspring.

**Methods:**

Lymphocytes were isolated from umbilical cord blood of infants born to 87 women with GDM and 81 women with normal pregnancy. Genomic DNA was extracted and DNA methylation levels of *GNAS* and *IGF2* DMRs were determined by Massarray quantitative methylation analysis.

**Results:**

The methylation levels were detected in 7 CpG sites of *GNAS* DMRs and 6 sites of *IGF2* DMRs. Methylation levels were significantly higher at sites 4, 5 and 7 of *GNAS* DMR in GDM compared to normal pregnancy (P = 0.007, 0.008 and 0.008, respectively). The methylation level at site 4 of *GNAS* was significantly correlated with the presence of GDM (P = 0.003), the methylation levels at site 5 and 7 were significantly correlated with the presence of GDM (P = 0.002 for both) and gestational age (P = 0.027 for both). There was no significant difference in any sites of *IGF2* DMR (P > 0.05 for all).

**Conclusions:**

We concluded maternal GDM-induced hypermethylation at *GNAS* DMR and this condition may be among the mechanisms associating maternal GDM with increased risk of metabolic diseases in later life of offspring.

## Background

Gestational diabetes mellitus (GDM), defined as any degree of glucose intolerance with onset or first recognition during pregnancy, affects 2 - 12% of all pregnancies [1,2]. GDM is associated with adverse pregnancy outcomes, including macrosomia and subsequently birth trauma, and fetal hypoglycemia, hypocalcaemia, respiratory distress, and even stillbirth, whereas treatments of GDM improve pregnancy outcomes [3,4]. GDM poses life-long risk to the offspring, such as increased birth weight, higher BMI at age of 6–24 and incidence of adolescent obesity compared to controls [5-7]. Numeral studies have shown an increase in metabolic syndrome in offspring of GDM pregnancies [8]. The prevalence of impaired glucose tolerance is higher in the offspring of mothers with gestational and pre-gestational diabetes than those of background population [9-12]. Animal studies revealed metabolic imprinting of intrauterine diabetic environment can be transmitted across generations [13]. Although studies in human and animal have demonstrated the association of GDM and risk of obesity, diabetes and metabolic diseases in offspring of GDM pregnancies, the mechanisms behind remain elusive.

Epigenetic modification might be among the mechanisms underlying this association [14]. The persistent epigenetic change in imprinted genes induced by prenatal environment may be among the mechanisms contributing to the association between maternal GDM and health problems in later life of offspring. Imprinted genes play important roles in embryonic growth and development and epigenetic disruption due to adversity in early life may be associated with susceptibility to metabolic diseases [15]. GDM induces differential methylation of genes in fetal DNA from offspring born to GDM mothers. Ruchat et al. [16] reported that GDM has epigenetic effects on genes preferentially involved in the metabolic diseases pathway and proposed that DNA methylation is involved in fetal metabolic and developmental programming. Differential methylation of fetal MEST and ABCA1 was also observed [17,18].

Insulin-like growth factor 2 (*IGF2*) is a paternally expressed gene widely expressed during prenatal development and its activity is regulated by genomic imprinting. Imprinted expression of *IGF2* is partially maintained through the differentially methylated region (DMR) located between exon 2 and 3, the disrupted methylation of which is associated with congenital growth disorders [19,20]. Guanine nucleotide binding protein alpha subunit (*GNAS*) is an imprinted gene with a highly complex imprinted expression pattern, which gives rise to maternally, paternally, and biallelically expressed transcripts [21,22]. The imprinted expression of *GNAS* is controlled by the DMR in exon 1A [23]. The methylation at differentially methylated region (DMR) of imprinted genes is established before gastrulation and is very sensitive to early developmental environment, but can be relatively stable throughout the life of individual [21,22]. The investigations in subjects conceived during Dutch famine winter revealed that the exposure to prenatal famine resulted in the persistent alteration in DNA methylation of imprinted genes including *GNAS* and *IGF2* [24,25]. Small-for-gestational age (SGA) and preeclampsia, conditions characterized by poor nutrient supply to fetus, induces hypomethylation at *IGF2* and *GNAS* DMRs and altered methylation at DMRs of imprinted genes may subsequently contribute to the development of metabolic diseases in later life [22,26-28]. However, the effect of GDM, a condition apposite to SGA and preeclampsia, on the methylation at DMRs of *IGF2* and *GNAS* of fetus has not been described yet.

In addition, although both of *GNAS* and *IGF2* gene are associated with obesity [29,30] and hypertension [31-34], currently there is no evidence if GDM modifies epigenetic imprints of *GNAS* and *IGF2* of fetus, or, *GNAS* and *IGF2* are possibly involved in the induction of high risk of GDM-related metabolic diseases in adulthood. Therefore, we determined fetal methylation levels at *GNAS* and *IGF2* DMRs of GDM and normal pregnancy in this study, and found increased methylation at *GNAS* DMR in fetuses of GDM compared to normal pregnancy.

## Methods

### Subjects

Eighty-seven women of GDM and 81 normal pregnant women were recruited in Women’s Hospital, School of Medicine, Zhejiang University, Shaoxing Women and Children’s Hospital, Ningbo Women and Children’s Hospital, Huzhou Maternity and Child Care Hospital and Jiaxing Maternity and Child Care Hospital.

Pregnancy was diagnosed upon positive human chorionic gonadotropin (hCG) test after missed menstruation. Gestational age was calculated by menstrual dating. Ultrasound was performed to confirm pregnancy and gestational age. GDM was diagnosed according to the criteria recommended by International Association of Diabetes and Pregnancy Study Groups (IADPSG) [35]. All GDM women controlled their glycemia with dietary control and physical activity. Exclusion criteria were multiple gestation, diabetes mellitus, chronic hypertension, infectious diseases recognized in pregnancy, premature rupture of membrane, active labor, polyhydramnios and signs of other concurrent medical complication. The control women had no sign of gestational complications and fetal distress and gave birth to healthy neonates of appropriate size for gestational age.

Clinical data and demographic data were collected according to the medical records. The approval of the current study was obtained from Institutional Ethical committee of Women’s Hospital, School of Medicine, Zhejiang University, and all the participants provided their informed consents.

### DNA methylation analysis

Umbilical cord blood samples were collected in Ethylene Diamine Tetraacetic Acid (EDTA)-treated tubes at delivery. Lymphocytes of infants were isolated and stored at -80C until use. Total DNA was isolated from lymphocytes using buffer ATL, proteinase K, and RNase A (Qiagen, Inc., Valencia, CA) followed by phenol–chloroform extraction and ethanol precipitation. Bisulfite conversion of DNA was carried out using the Epitect Bisulfite Kit (Qiagen Inc., Valencia, CA).

Quantitative methylation analysis of DNA was performed using MassARRAY EpiTYPER assays (Sequenom, San Diego, CA) according to the protocol recommended by the manufacturer. Methylation of all CpG sites at the *IGF2* DMR in Chr11: 2169100–2169551, and *GNAS* DMR in Chr 20: 57,415,713-57,416,072 was measured, quantitative methylation of each CpG unit was calculated following the method described in our previous study [28]. Seven CpG sites in the *GNAS* DMR and six CpG sites in the *IGF2* DMR were qualified for reliable detection based on CpG unit mass quality evaluated by EpiTYPER and were used for the further statistic analysis. The other CpG sites in the DMRs were discarded as they are either non-quantifiable or ambiguous CpG sites. Positions of each CpG site was stated in our previous study [28].

### Statistical analysis

The Kolmogorov-Smirnov test was used to evaluate the distribution of data. Continuous data were presented in mean and standard deviation (SD) and compared with Student *t*-tests while categorical data were evaluated with Chi-square test. Multiple variant regression analysis was used to evaluate the relationship of methylation level with the presence of GDM, birth weight, maternal age, gestational age at delivery and fetal gender. SPSS statistical package (Statistical Analysis System, Chicago, IL) was used for the statistic analysis. Values of P < 0.05 were considered to be statistically significant.

## Results

As shown in Table 1, there were significant differences in maternal age, gestational age at delivery and neonatal birth weight between GDM and normal pregnancy (P < 0.001 for all). Maternal age was greater, gestational age was shorter and birth weight was heavier in GDM group compared to normal pregnancy. There was no significant difference in fetal gender (P = 0.504).

**Table 1:** **Clinical data and mean methylation levels at** ***GNAS*** **and** ***IGF2*** **DMRs**

	**Normal pregnancy**	**Gestational diabetes**	**Significance**
n	81	87	
**Maternal characteristic**			
Maternal age (y)	28.6 ± 3.4	30.61 ± 3.8	<0.001
Gestational age at delivery (w)	38.70 ± 1.37	37.56 ± 1.86	<0.001
**Infant characteristics**			
Birth weight (g)	3288 ± 395	3559 ± 592	<0.001
Fetal gender	Male:41	Male:45	0.504
	Female:40	Female:42	
**Methylataion data**			
methylation level at *GNAS* DMR	0.51 ± 0.056	0.52 ± 0.059	0.271
methylation level at *IGF2* DMR	0.46 ± 0.043	0.46 ± 0.048	0.689

**Figure 1:**
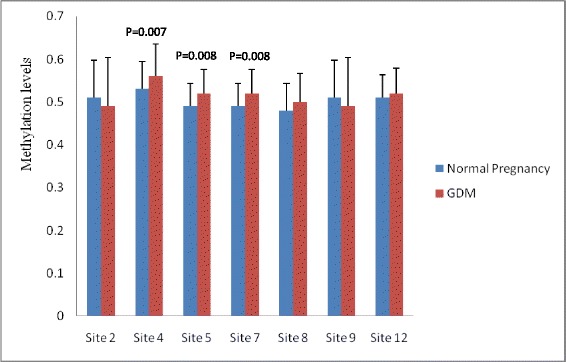
**Site-specific methylation levels at** ***GNAS*** **DMR in fetuses of normal pregnancy and gestational diabetes (GDM).** There were significant differences in methylation levels at site 4, 5 and 7 between fetuses of normal pregnancy and GDM (P = 0.007, 0.008 and 0.008, respectively). The differences were not significant in the methylation levels at site 2, 8, 9 and 12 (P = 0.254, 0.122, 0.254 and 0.077, respectively).

**Figure 2:**
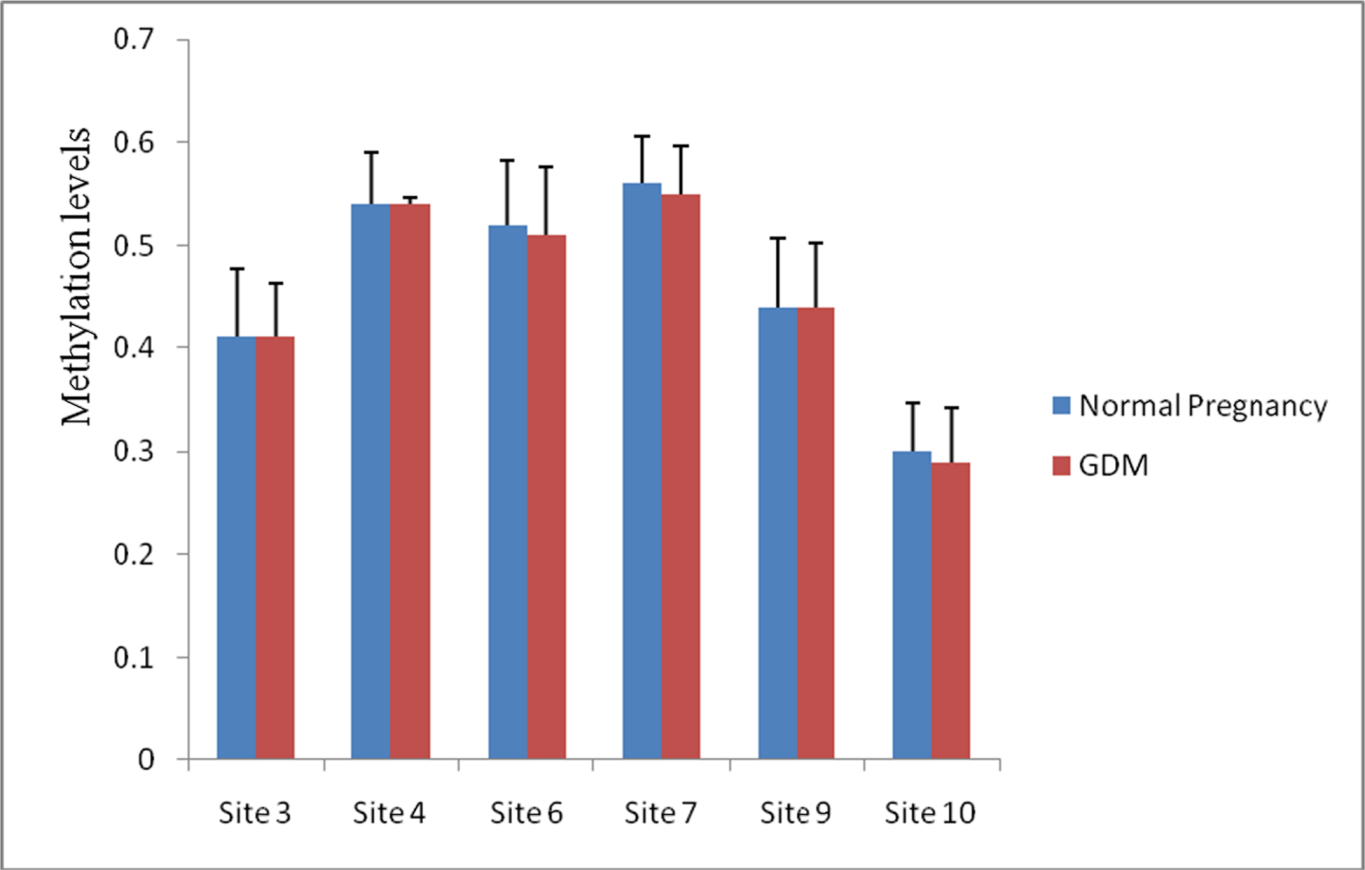
**Site-specific methylation levels at** ***IGF2*** **DMR in fetuses of normal pregnancy and gestational diabetes (GDM).** There were no significant differences in methylation levels at sites 3, 4, 6, 7, 9 and 10 of *IGF2* DMR between GDM and normal pregnancy (P = 0.792, 0.840, 0.455, 0.784, 0.845 and 0.214, respectively)

The methylation levels were detected in 7 CpG sites of *GNAS* DMRs and 6 sites of *IGF2* DMRs (Figures 1 and 2). Methylation levels at sites 4, 5 and 7 of *GNAS* DMR were significantly higher in GDM compared to normal pregnancy (P = 0.007, 0.008 and 0.008, respectively), but there were no significant differences in methylation levels at sites 2, 8, 9 and 12 (P = 0.254, 0.122, 0.254 and 0.077, respectively). The methylation level of CpG site 4, 5 and 7 of *GNAS* DMR were significantly correlated with the presence of GDM (P = 0.003; P = 0.002 for site 5 and 7), furthermore, the methylation levels of sites 5 and 7 were significantly correlated with gestational age (P = 0.027 for both).

There were no significant differences in methylation levels at CpG sites 3, 4, 6, 7, 9 and 10 of *IGF2* DMR between GDM and normal pregnancy (P = 0.792, 0.840, 0.455, 0.784, 0.845 and 0.214, respectively). When it comes to the mean methylation levels of all those CpGs sites at the *GNAS* and *IGF2* DMRs, there were not significantly different between GDM and normal pregnancy (P = 0.271 and 0.689, respectively)(Table 1).

## Discussion

In the current investigation, increased methylation in CpGs sites of *GNAS* DMR was observed in fetuses of GDM women compared to control. Our data imply that increased methylation at *GNAS* DMR may be among the mechanisms linking maternal GDM with high risk of metabolic diseases in later life of fetuses. Whereas, unchanged methylation at *IGF2* DMR indicate *IGF2* is not vulnerable to GDM-induced intrauterine environment.

*GNAS*, the gene encoding G protein alpha (GSα), is imprinted in a tissue-specific manner. There are three DMRs located from upstream to downstream of the GSα promoter: the first one is in the NESP55 promoter region, the second is in the *NESPAS/GNASXL* promoter region and the third one is located in the *GNAS* exon 1A region. The Exon 1A DMR controlls the imprinted expression of *GNAS* [20]. GSα is equally expressed from both alleles in most tissues, but is expressed primarily from the maternal allele in certain hormone-target tissues, such as thyroid and pituitary et al. [36,37]. The DMR analyzed in our study, which is located in the exon 1A DMR, is necessary for tissue-specific imprinting of GSα [23]. GSα plays an important role in energy metabolism and the development of obesity [29]. The mutations of *GNAS* which disrupt GSα expression or function cause Albright hereditary osteodystrophy (AHO), a congenital syndrome which is characterized by obesity, short stature et al. [29,30]. Patients of AHO with mutation located on the maternal allele develop resistance to hormones including parathyroid hormone, thyrotropin, growth hormone-releasing hormone and gonadotropins. In this condition, obesity occurs very early (usually within the first year) and tends to be severe in early childhood. In addition, animal study showed that *GNAS* knock-out mice with an insertion mutation on maternal allele develops obesity with increased serum leptin levels and lipid accumulation [38].

It was reported that prenatal exposure to adverse environment, such as disturbed intrauterine environment, dysregulates the fetal epigenome, with potential consequences for subsequent developmental disorders and disease over the life course [39]. However, no literature describing the effects of GDM on fetal *GNAS* was available yet. Herein, we reported for the first time the increased methylation at *GNAS* DMR in GDM fetuses compared to control, indicating methylation of *GNAS* DMR is sensitive to GDM-induced intrauterine environment. Regression analysis excluded the confounder effect of maternal age and birth weight on methylation in GNAS DMR but we could neither exclude the possible effect of gestational age nor determine the causal relationship between GDM and hypermethylation of GNAS DMR. As disruption in the imprinted expression of GSα leads to the development of obesity, our data also provide a clue to mechanisms linking maternal GDM with risk to chronic diseases for infants in adulthood, such as obesity. Further evidence linking increased methylation at *GNAS* DMR with the expression or function of GSα in a tissue-specific manner would enhance our insight into the role of increased methylation at *GNAS* in the development of obesity. Animal models or cell lines are needed for these purposes.

In this paper, we also analyzed differences of methylation levels in the *IGF2* DMR in fetuses of GDM women comparing to control. The imprinting of *IGF2* is maintained by two regulatory DMRs located close to exon 3 (also referred to *IGF2* DMR or DMR0) and upstream of neighboring maternally expressed *H19* (referred to *H19* DMR). Multiple CpG sites in the *IGF2* DMR were found to be associated with higher newborn birth and placenta weight, while no significant association was observed in the *H19* DMR [40]. In addition, hypomethylation of *IGF2* DMR was also associated with elevated plasma *IGF2* protein concentrations and higher birth weight in infant born to obesity women while patterns of association were not apparent at the *H19* DMR [41]. Another study revealed that the methylation levels of *IGF2* DMR were affected by folic acid intake before or during pregnancy and depression in pregnancy [42]. Taken together with our previous study reported significant lower methylation in offspring of preeclampsia than normal pregnancy [28], it indicates that the methylation of *IGF2* DMR could be a better biomarker than *H19* DMR to evaluated the effect of environmental exposure in early development. Therefore, we investigated the association between methylation levels of the *IGF2* DMR located between the exon 2 and 3 of *IGF2* with intrauterine exposure to GDM. However, our study failed to find significant methylation changes of *IGF2* DMR between infants born to GDM mother and normal control. The lack of association between the *IGF2* DMR methylation profile and GDM in our study indicated that the *IGF2* DMR may not be vulnerable to GDM-induced intrauterine environment.

However, animal studies revealed hypermethylation at IGF in diabetic offspring. Shao et al. reported that the methylation level of the *H19–Igf2* imprint control region was 19.1% higher while the body weight was 26.5% lower in pups born to diabetic mice compared to controls [43]. Ding et al. observed hypermethylation at *H19-Igf2* DMRs and reduced expression of *IGF2* in pancreatic islets isolated from pups of diabetic mice [44]. In addition, murine diabetes influenced placental expression of *Igf2* [45]. Murine diabetes is induced by intraperitoneal injection of streptozotocin and is characterized by poor maternal nutritional condition and low pup weight. The birth weight is usually higher in GDM than control as demonstrated in the current investigation. The differences in maternal disorder (GDM versus streptozotocin-induced diabetes), maternal nutrition condition (good versus poor), species (human versus murine) and birth weight are the possible factors responsible for the different methylation in *IGF2* MDR observed in humans and mice.

## Conclusion

In summary, we reported that GDM induced significantly increased methylation levels at fetal *GNAS* DMR compared with control, suggesting that hypermethylation at *GNAS* DMR may be among the mechanisms linking maternal GDM with high risk for chronic diseases in later life of offspring. Further investigation is needed to clarify the exact role of *GNAS* in the development of obesity and hypertension and to identify possible target point for the reduction of the risk for metabolic diseases in later life of GDM fetuses.

## Abbreviations

*IGF2*: Insulin-like growth factor-2
*GNAS*: Guanine nucleotide binding protein, alpha stimulating
DMR: Differentially methylated region
DNA: Deoxyribonucleicacid
EDTA: Ethylene Diamine Tetraacetic Acid
SGA: Small for gestational age
AGA: Appropriate for gestational age
IADPSG: International Association of Diabetes and Pregnancy Study Groups
PCR: Poly-chain reaction
